# Shedding Light on Roman Glass Consumption on the Western Coast of the Black Sea

**DOI:** 10.3390/ma15020403

**Published:** 2022-01-06

**Authors:** Roxana Bugoi, Alexandra Ţârlea, Veronika Szilágyi, Ildikó Harsányi, Laurenţiu Cliante, Irina Achim, Zsolt Kasztovszky

**Affiliations:** 1 Horia Hulubei National Institute for Nuclear Physics and Engineering, 30 Reactorului Street, 077125 Măgurele, Romania; 2 Faculty of History, University of Bucharest, 030018 Bucharest, Romania; alexandra.tarlea@istorie.unibuc.ro; 3 Centre for Energy Research, 29-33 Konkoly-Thege Street, H-1121 Budapest, Hungary; szilagyi.veronika@ek-cer.mta.hu (V.S.); harsanyi.ildiko@ek-cer.mta.hu (I.H.); kasztovszky.zsolt@ek-cer.mta.hu (Z.K.); 4 Museum of National History and Archaeology, 12 Piaţa Ovidiu, 900745 Constanţa, Romania; cliante@gmail.com; 5 Vasile Pârvan Institute of Archaeology, 11 Henri Coandă Street, 010667 Bucharest, Romania; irina.adriana.achim@gmail.com

**Keywords:** Roman glass, PGAA, Histria, Tomis, chemical composition, Early Roman Empire

## Abstract

The chemical composition of 48 glass finds from Histria and Tomis, Romania, chiefly dated to the 1st–4th c. AD, was determined using prompt gamma activation analysis (PGAA) at the Budapest Neutron Centre (BNC). Most fragments have composition typical for the Roman naturally colored blue-green-yellow (RNCBGY) glass; Mn-colorless, Sb-colorless, and Sb–Mn colorless glass finds were evidenced, too. Several Foy *Série 2.1* and Foy *Série 3.2* glass fragments, as well as an HIMT and a plant ash glass sample, were identified in the studied assemblage. The archaeological evidence, the glass working waste items, and the samples with compositional patterns suggestive of recycling are proofs of the secondary glass working activities at Tomis during the Early Roman Empire period.

## 1. Introduction

Compositional analyses of archaeological glass finds can provide insights into raw materials and working techniques, demonstrating the skills of our predecessors [1]. Compositional data can also shed light on the glass provenance or manufacturing practices [2].

The archaeometric research on Roman glass finds evidence remarkably similar compositional patterns, especially during the Early Roman Empire period [3,4,5].

Roman glass is mostly identified as soda–lime–silica glass, typically containing 63–75 wt% silica (SiO_2_), 11–22 wt% soda (Na_2_O), and 4–12 wt% lime (CaO). Flux (the soda component) was introduced in the recipe to allow the melting of the mixture at temperatures attainable in ancient furnaces, while the stabilizer (lime) was needed to make glass stable in the presence of water, as pure soda–silica glass would be soluble in aqueous solutions.

In ancient times, two major sources of soda were available to the glass workers from Europe, North Africa, and the Near East: the ashes of halophytic plants and the mineral natron. “Natron” is a term used in archaeology to describe the deposits of salts containing sodium carbonate minerals, along with chlorides and sulfates that formed through the evaporation of soda lakes, mostly in Wadi Natrun in Egypt. However, east of the Euphrates, in the Persian Empire, plant ash was used as a soda source in glass making without any interruption during the 1st millennium AD [2].

During the Roman period, glass was fused in a restricted number of primary workshops located on the Egyptian or Syro-Palestine shores of Mediterranean Sea. It is worth mentioning here the remarkable tank furnaces discovered in the Levant and in Egypt [6,7,8], in which huge blocks of raw glass were made from carefully selected calcareous sands (i.e., shell-containing sands from particular beaches) and the mineral flux natron originating from Egypt [2].

From these primary workshops, glass was shipped via maritime routes, preferably as small pieces and just occasionally as finite products, toward the so-called consumption places spread all over the Roman Empire.

Of special importance for the history of glass was the discovery of several Roman shipwrecks with glass chunks as cargo, such as the *Embiez* (2nd c. AD), which contained lumps of fresh glass, as well as finished vessels and windows [9,10], and the *Iulia Felix* (3rd c. AD), which carried a barrel with fragments of glass bottles, plates, and cups collected for recycling [11].

A large number of small workshops scattered all over the empire and also in the regions under the influence of Rome dealt with the manufacturing of finished objects: vessels, window panes, and adornments. The basic raw material for these secondary workshops consisted of glass chunks dispatched from the primary workshops. As in many other historical periods, recycling was often encountered in secondary glass making, involving the collection, melting, and mixing of broken vessels or discarded adornments. When glass was recycled and reused, the color criteria were also taken into account [12].

During the Roman period, the manipulation of glass color was quite sophisticated. In particular, colorless glass was highly appreciated. However, items of intentionally colored glass were also occasionally encountered in archaeological records: beads, bangles, colored vessels, and decorative features (stripes, blobs, and handles) applied on naturally colored vessels.

In principle, Roman glass contains ≥0.3 wt% Fe_2_O_3_, the iron component being mainly inherited from the glassmaking sands. This accidentally incorporated iron provides glass a distinctive blue or green-blue tint. The typical aqua blue color of Roman glass is due to iron in reduced state (Fe^2+^ cation), while the fully oxidized state iron (Fe^3+^ cation) leads to a pale-yellow color. The typical green hue of Roman glass is a consequence of a relatively well-balanced mixture of the two cations of iron [2].

Antimony minerals are powerful decolorizers that were preferentially employed in Roman glass making until the 4th c. AD. Thus, the addition of antimony compounds to the glass batch led to the removal of the faintest tints of blue, green, or yellow and upon analyzing it, the resulting glass was classified as Sb-colorless. The extensive use of this particular additive coincided with the spread of glass blowing across the Roman Empire.

The competitor decolorizers were the manganese compounds; however, in order to produce a similar effect on glass appearance, manganese compounds had to be added in higher amounts (~1 wt% MnO). This decolorizer prevailed especially during the Late Roman Empire period; however, its occasional presence in earlier artefacts is also attested in the archaeometric literature [13].

The most-often employed glass chromophores in ancient times were the ions of particular metals: copper, cobalt, tin, antimony, lead, manganese, and iron. One of the most remarkable is cobalt, an element that even if present in extremely low amounts (some hundreds of parts per million) would induce a typical deep-blue hue to the glass. Copper in the upper oxidation state would impart glass a completely different turquoise blue or green hue, depending on the presence of other elements, such as iron or lead, while copper in a reducing state would lead to the production of the highly prized opaque red glass. Other chromophores used in Roman glass making were iron (for black) and manganese (brown or violet). To summarize, intentionally colored glass resulted from the interplay of the oxidation states of particular metallic ions and the concentrations at which various chromophore compounds were encountered in the glass batch [2].

Roman glass artefacts discovered at archaeological sites on the western coast of the Black Sea and in the Lower Danube region have been rarely investigated from compositional point of view [14,15,16,17,18]. The chemistry of ancient glass finds excavated in Romania started to be systematically studied only during recent years [19,20,21,22,23].

This paper reports the chemical composition of 48 glass fragments excavated at Histria (33 samples) and Tomis (15 samples), Romania, mostly dated to the Early Roman Empire period (1st–3rd c. AD). A small number of samples (six fragments discovered at Histria) turned out to be actually dated to the Late Roman Empire period (5th–6th c. AD). The measurements were performed at the Budapest Neutron Centre (BNC), using a bulk non-destructive analytical technique, namely prompt gamma activation analysis (PGAA).

The main aim of this study was to assign the analyzed fragments to well-established chemical groups of Roman glass from the archaeometric literature [3,5,13,24,25,26] to ease the comparison with data on coeval finds discovered in other regions of the Roman Empire. This classification was subsequently used to draw some conclusions about the provenance of glass from which these vessels were shaped, as well as on the manufacturing techniques.

This study is part of a larger project targeting to unravel the story of the circulation and consumption of glass in the Black Sea zone during the Roman period, in a trial to integrate this area with the impressive corpus of chemical data on Roman glass existing in the archaeometric literature.

## 2. Materials and Methods

### Archaeological Background and Analyzed Samples

**Figure 1:**
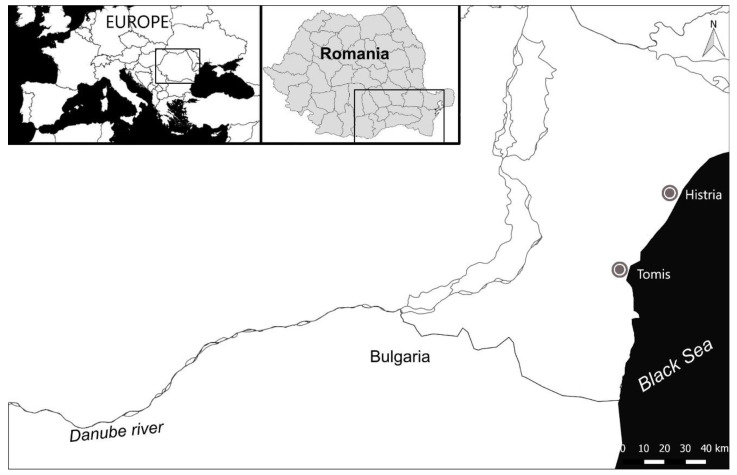
Maps showing the location of the archaeological sites where the glass fragments reported in this paper were discovered.

Histria (Istros) is one of the most significant archaeological sites of Romania. Founded on the western coast of the Black Sea by the Ionian Greek city of Miletus, Histria played an important political and economic role from the end of the 7th c. BC until the beginning of the 7th c. AD (see Figure 1). After its integration into the Roman Empire during the last half of the 1st c. BC, Histria became a part of the Roman production and trade network [27,28].

Different types of glassware are well represented in the archaeological record from Histria, as intact vessels, but more often as fragments [29,30,31,32,33].

So far, no glass working kilns or other indicators of glass manufacturing activities, such as crucibles or glass working waste fragments, have been discovered at Histria. However, taking into account the abundance of glass finds—and one should mention here the 1600 lamps and goblets (mostly Isings 111 forms) discovered in the basilica from Histria and published in [29,30]—the local production of glass vessels at Histria during the Late Roman Empire was postulated.

A project supported by the University of Bucharest (2013–2016 and 2017–2021) led to the opening of a new excavation sector at Histria, named Sector Acropola Centru-Sud/Acropolis Center-South (ACS) Sector, which revealed a 6th c. AD building [34,35].

The Crypt Basilica Sector, informally called the Basilica Florescu (BFL) Sector, situated in the central-northern part of the Late Roman/Early Byzantine city, has been excavated, with some interruptions, since 2002. The research is conducted by the Vasile Pârvan Institute of Archaeology, Bucharest, and has as the main objective the restoration of the Christian monument, based on a better understanding of the topographic situation and of the archaeological contexts from the entire area [34,36,37].

Tomis, another significant archaeological site of Romania, is also situated on the western coast of the Black Sea, approximately 50 km south of Histria. Its remains are practically located under the city of Constanţa (see Figure 1). It was a colony of the Greek city of Miletus, too, being founded during the second half of 6th c. BC. Tomis came under Roman control at the same time as Histria and enjoyed a flourishing economic life during the Early Roman Empire period (1st–3rd c. AD), being the largest city of Moesia Inferior province [38].

The excavations conducted at Tomis during the 20th and 21st c. led to the discovery of a large number of Early Roman Empire glass finds (jugs, cups, decanters, and *unguentaria*) in well-dated contexts, especially in graves [39,40,41,42,43], as well as several kilns for glass working [32,39,44].

Because the finds related to glass production were the result of rescue excavations in specific conditions, i.e., incomplete excavations or archaeological contexts affected by the subsequent interventions, as the modern city of Constanţa superposes upon the ancient city of Tomis, most of them were either not published or just mentioned in the literature, without providing too many details [44]. Consequently, for the time being, the knowledge on glass working activities at Tomis during the Early Roman Empire period is rather sketchy.

During the Roman period, the import of glass vessels represented an important part of the economic exchanges between the western coast of the Black Sea and the Mediterranean zone [29,30,32].

So far, six (possibly seven) glass kilns and remains related to secondary glass working have been identified in Tomis [32,39,44]. These finds have been loosely dated to the 1st–4th c. AD. All these glass kilns are of small size and circular shape, consistent with the secondary production of glass items relying on the use of imported raw glass and recycled cullet; most likely, they manufactured vitreous items for the local and regional market. These discoveries confirm the hypothesis of local glass working at Tomis during the Early Roman Empire period. Most likely, Tomis was a supplier of glass objects for the neighboring cities, too—e.g., Histria.

The large number of glass vessels of various types, differing greatly in shape, size, and quality, that were discovered in funerary contexts at Tomis, mostly dated to the 1st–3rd c. AD, brought further support to the idea of local glass working.

From this perspective, three vessels with globular bodies and long necks, identified as grave goods, are particularly important as they had the moil undetached at the moment of their deposition in the graves [32,39,40,44].

To get some hints about glass consumption during the Early Roman Empire period in these two cities on the western coast of the Black Sea, an archaeometric study was initiated, targeting the compositional analysis of 48 glass fragments. This study intended to provide chemical data that would allow speculating about the raw materials and the provenance (i.e., the place where glass was fused from raw materials) and also about the way these glass items were made, i.e., manufacturing techniques, for example, whether there was any recycling involved in their making.

Twenty-nine glass fragments from Histria were analyzed using the PGAA technique at BNC in April 2019. They included the following: 14 vessel fragments, out of which 5 dated to the 1st–3rd c. AD and 9 dated to the 4th–6th c. AD, discovered in the layer of the Late Roman building in the ACS Sector, and 15 vessel fragments from the 1st–3rd c. AD, unearthed in the BFL Sector.

This selection of fragments aimed the determination of the chemical composition of coeval glass fragments excavated at Histria in slightly different contexts. Thus, the BFL Sector offered the opportunity of recovering glass fragments from a context dated to the 1st–3rd c. AD, while in the ACS Sector, the Early Roman Empire glass fragments were excavated from the 6th c. AD layer, the last inhabitation phase of the city; in particular, the items analyzed in this study were discovered in secondary positions.

For comparison, 19 glass samples from Tomis (14 vessel fragments and 5 glass working waste items discovered in two kilns), dated to the 1st–4th c. AD, were measured as well. Fragments from two glass vessels excavated from a dated funerary context (3rd–4th c. AD), namely Tomis-2 and Tomis-3, are of particular interest, as they are unfinished products, i.e., the moil is still undetached from the rim, suggesting that they were locally manufactured with the intention to be deposited as grave goods [44].

Given the fact that some finds belong to the same type, the archaeologists also wanted to check for eventual similarities in their chemical composition; this particular point will be tackled in the Results and Discussion section.

All these glass fragments offer the advantage of having been discovered in well-defined archaeological contexts. Moreover, they can be considered, up to a certain point, representative of the repertoire of Early Roman Empire vessels unearthed at Histria and Tomis (thousands of such items: some intact glass vessels, but mostly fragments).

**Figure 2:**
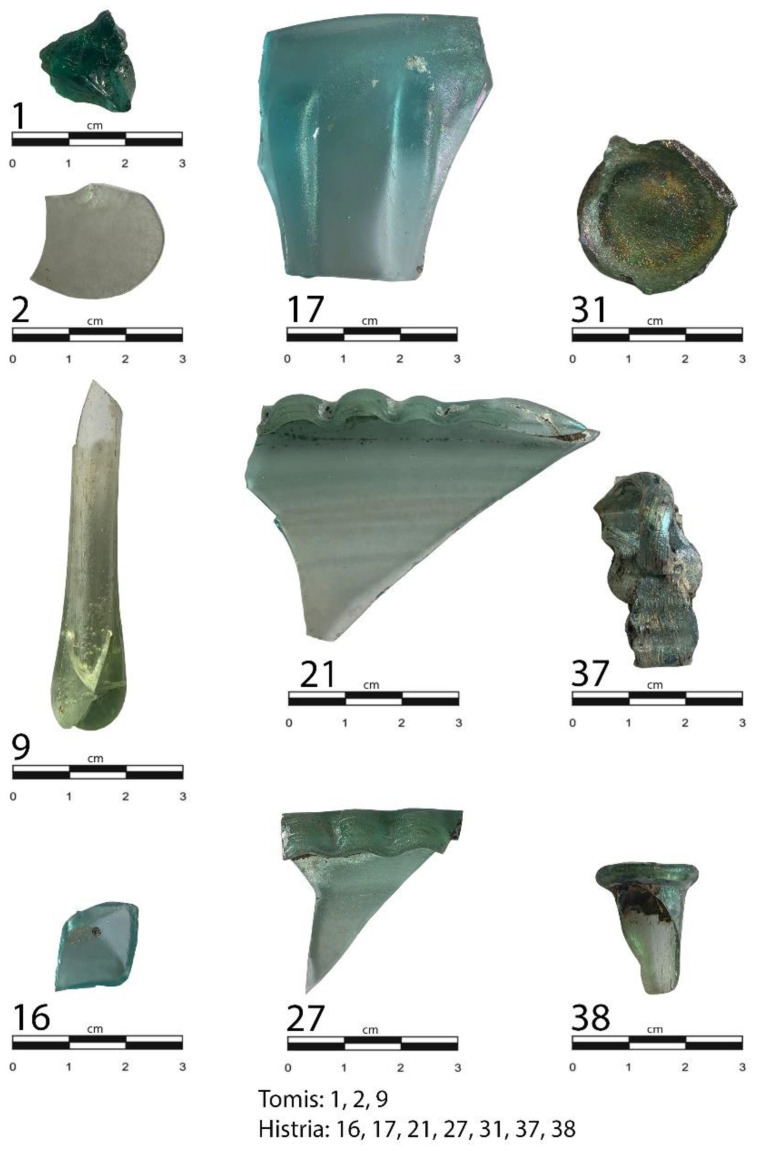
Photos of selected glass samples reported in this study.

The detailed typological and chronological classification of the analyzed samples, made according to stylistic and stratigraphic criteria, is given in Table S1 and illustrated by Figure 2.

This study aimed to identify the finds made of fresh glass and to distinguish, if possible, the fragments whose composition would suggest their manufacture using recycled glass. In particular, the compositional data were used to provide additional, circumstantial evidence for the hypothesis that local glass working activities took place in this region during the Roman period.

## 3. Experimental

The bulk elemental composition of the glass samples was determined at the Budapest PGAA facility. The present setup of the BNC facility is described in detail in [45]. The concentrations of different chemical elements are determined using the detection of gamma rays emitted during (n, γ) reactions.

The method is applicable for the quantitative determination of the bulk elemental composition, comprising the major components and some minor and trace elements, in particular, to measure the concentrations of those chemical elements with high-neutron-absorption cross sections.

In PGAA, quantitative results are determined using the prompt-k_0_ method, which is an internal standardization method [46,47]. The standardization of PGAA at BNC has been published in [48,49].

Because of the high penetration of neutrons, the technique provides the average composition of the irradiated volume (usually on the order of a few centimeter cubes). Thanks to the relatively low intensity of the neutron beam, the induced radioactivity decays quickly after the irradiation and the artefacts can be safely returned to the owners. PGAA is well suited for the study of cultural heritage objects since this method does not require any sample preparation or extraction of small samples from the artefacts, allowing the completely non-destructive characterization of unique archaeological finds.

The PGAA facility from BNC has been extensively used to analyze various kinds of cultural heritage artefacts, including ancient glass items [15,17,50,51,52,53].

The accuracy and precision of the PGAA setup from BNC has been checked by repeated measurements of several certified glasses; for details on this issue, refer to [52,53]. In particular, the PGAA results on Corning Museum of Glass archaeological reference glasses Brill B and Brill C are provided in Table S2, along with some published values [54].

According to [55], when applying the k_0_ method in quantitative PGAA, the most significant source of uncertainty for the concentration values is related to the peak area determination, i.e., counting statistics. Therefore, it was not necessary to perform repeated measurements on each sample.

A total of 48 samples, namely 42 glass fragments originating from different types of vessels, 1 bead, and 5 glass waste chunks, were analyzed during the PGAA experiment from BNC.

The glass samples were irradiated with an external cold neutron beam of 9.6 × 10^7^ cm^−2^ s^−1^ thermal equivalent intensity for 1700–20,000 s. The acquisition times were optimized to collect gamma spectra with statistically significant peaks for the elements of interest. The cross section of the external neutron beam varied between 24 mm^2^ and 400 mm^2^; thus, in most cases, the entire glass fragment was exposed to the neutron beam.

The prompt-gamma spectra were collected using a 64 k MultiChannel Analyzer, and the Hypermet-PC software was used for spectrum evaluation. The quantitative analysis is based on the k_0_ principle using a PGAA library developed at the BNC [49].

For radiation safety reasons, in particular because of the activation of Na (the half-life (T_1/2_) of ^23^Na is 14.96 h), it was mandatory to keep the glass samples in the laboratory for 2–3 more days after the experiment to allow the decay of the ^23^Na nuclei.

Before the PGAA experiment, all glass fragments were washed with water and gently brushed to remove the superficial dirt and soil deposits; however, on several samples, despite the cleaning procedures, visible thick layers of weathering products remained firmly attached to the glass surface. In principle, the chemical composition of an ancient glass fragment that has been buried for centuries is altered to a depth of some tens of micrometers compared to the bulk composition. In these thin layers, the alkali, alkali earth elements, and silica concentrations are modified by leaching phenomena [56]. However, due to the high penetration of cold neutrons in tandem with the detection of gamma rays, the reported PGAA concentrations can be considered as representative of the chemistry of the pristine glass of all samples.

Using PGAA, it was possible to quantify H, B, Na, Al, Cl, Si, Ti, K, Ca, Mn, Fe, Sm, and Gd in all glass fragments and Mg, S, Co, and Sb only in certain samples, where these elements are present above the detection limits. The data expressed in wt%, as well as the detection limits of the PGAA setup, are given in Table S3. The concentrations of all elements are expressed as oxides, except for Cl, which is given in elemental form. The overall uncertainties of the reported concentrations were estimated to be less than 10% (relative values) from the reported numbers.

## 4. Results and Discussions

All analyzed glass fragments were identified as soda–lime–silica glass fragments, as indicated by the average values of soda (17.2 ± 1.7 wt% Na_2_O), lime (6.9 ± 0.8 wt% CaO), and silica (68.4 ± 2.3 wt% SiO_2_).

The magnesia and potash content for most samples is less than 1.5 wt%, a consequence of the fact that natron was the mineral flux used in making these glass items [57]. While potash was quantitatively determined in all samples, in some of them, magnesia is under the detection limits of the PGAA setup, estimated to be 1.0 wt% MgO.

However, several samples (Tomis-15, Tomis-48, Histria-17, Histria-31, Histria-38, and Histria-39) feature magnesia concentrations slightly higher than the 1.5 wt% value that in the literature separates the natron glass from that made of plant ashes [57].

In particular, sample Histria-39 has both magnesia and potash content higher than 1.5 wt%. This finding was interpreted as indicative of a plant ash component in the composition of this glass fragment. Recycling procedures involving the addition of plant ash glass cullet to natron glass chunks might be a possible explanation for this particular compositional pattern. This idea is also supported by the chlorine content, which is the lowest in this sample set (0.66 wt% Cl), a further argument for recycling, taking into account that chlorine is a volatile element [58].

High hydrogen content (H_2_O concentrations varying from 0.9 up to 3.5 wt%) was detected in several fragments (Histria-23, Histria-36, Histria-39, Tomis-2, Tomis-9, Tomis-13, Tomis-14, and Tomis-15). Thick layers of soil and weathering products are visible on these particular samples, despite the cleaning with water performed before the PGAA experiment. The explanation might be the hydrated mineral compounds that still cover the surfaces of these glass fragments.

A summary of the glass groups identified in the studied assemblage is given in Table S3, including the mean values for larger groups, as well as some comparison terms from the archaeometric literature [3,5,13,24,26,59].

The glass fragments reported in this paper are either colorless or naturally colored; the detailed sample description is given in Table S1. Therefore, the first criterion taken into account when performing the classification of the samples into compositional groups was their content of chemical elements indicating the intentional addition of decolorizing compounds, i.e., Sb and Mn oxides. Further, the concentrations of major and some minor elements of the vitreous matrix (Si, Na, Ca, K, Mg, Ti, Fe, and Al) were considered as discriminating factors.

The fragment of a flask Histria-41 is a sample whose lack of color can be explained by its relatively high content of antimony (7500 ppm Sb_2_O_3_), accompanied by a small manganese concentration (320 ppm MnO), close to the limits given in the literature that would suggest the recycling and mixing of Sb-colorless with Mn-colorless glass, i.e., 250 ppm MnO [13]. The compositional pattern of this sample, with little alumina (1.68 wt% Al_2_O_3_), titanium (0.073 wt% TiO_2_), and lime (5.03 wt% CaO), indicates that mature sand was used to manufacture this glass, most likely of Egyptian origin [13]. Sb-decolorizing procedures were widespread during the Early Roman Empire period, being frequently used until the 4th c. AD, when a steep decline began [13].

Two other colorless samples from this assemblage might be also attributed to the Sb-colorless group: Tomis-11 and Tomis-15, containing relatively high amounts of antimony (8900 and 7800 ppm Sb_2_O_3_, respectively) and low amounts of manganese (440 and 460 ppm MnO, respectively), accompanied by low concentrations of lime (5.14 and 6.03 wt% CaO, respectively) and low concentrations of alumina (2.08 and 1.97 wt% Al_2_O_3_, respectively); these compositional patterns are characteristic for Sb-colorless glass finds. One should mention though that the values of the MnO concentrations are a bit higher those from the archaeometric literature, which indicates the exclusive use of antimony as a decolorizer, i.e., above the background level of MnO naturally contained in the sands used for making glass [13,60].

Nine colorless samples from the studied sample set owe their lack of color to a relatively high manganese content, or 1.62 wt% MnO on average (see Table S3), suggesting the prevalence in the studied sample set of colorless glass made using this a decolorizer; compare this with the three fragments discussed before, representative of the alternative technological solution, namely the use of antimony decolorizing compounds. Mn-colorless glass is often encountered in artefacts dated to the Late Roman Empire period, but this type of glass also existed during the Early Roman Empire period, though less frequently reported in the literature [13]. In all these samples, antimony, even if present, is under the detection limits, estimated to be approximately 1000 ppm Sb_2_O_3_ for the PGAA setup.

The samples from this group have different typological and chronological attributes, varying from the Early Roman Empire to the Late Roman Empire period. For example, samples Histria-33, Histria-43, and Histria-44 were dated to the 6th c. AD.

The overall inhomogeneity of this group, chronological, typological, and compositional, suggests that the raw glass from which these vessels were shaped might had originated in Levantine and Egyptian workshops [13].

Nine samples from the studied sample set fall into the group of the Sb–Mn colorless glass, being characterized by intermediate amounts of both decolorizers (~4000 ppm MnO and 4900 ppm Sb_2_O_3_ on average). Their compositional pattern is in good agreement with the values for the Roman Sb–Mn colorless glass from the literature (1st–4th c. AD) [5,13]; see Table S3. These samples are clearly the result of recycling procedures involving both types of colorless glass. It is worth mentioning that most of these fragments were found in excavations in Tomis (six out of nine samples).

**Figure 3:**
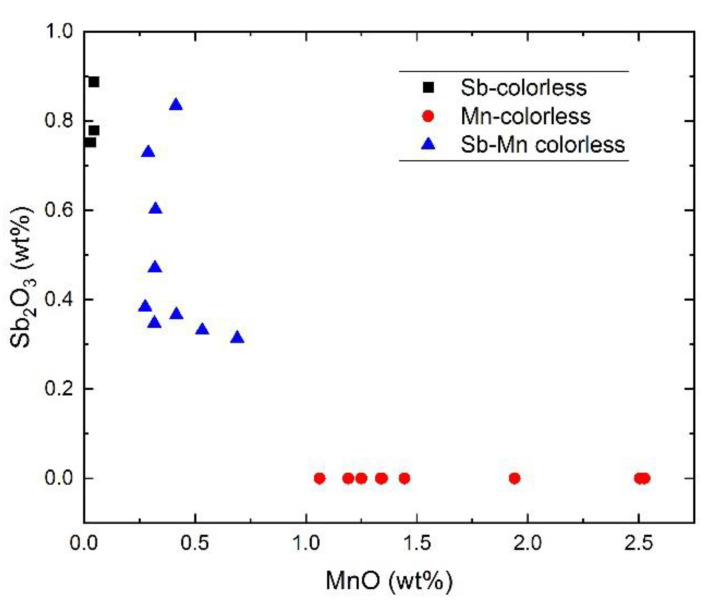
Plot of Sb_2_O_3_ versus MnO concentrations in the colorless glass samples from Histria and Tomis discussed in this publication.

The graph shown in Figure 3 is a good illustration of the division of the colorless samples into these three groups; in particular, it supports the attribution of samples Tomis-11 and Tomis-15 to the Sb-colorless group, despite their slightly high manganese content.

All these classifications were made considering the groups of colorless glass defined in [13].

Most of the naturally colored and colorless glass fragments (15 items) were attributed to the well-known chemical group of Roman glass from the literature, the so-called RNCBGY2 group defined by Gliozzo and colleagues (2016), where RNCBGY2 stands for Roman Naturally Colored Blue Green Yellow 2, thought to originate in some Levantine primary workshops [26]. Similar compositions, i.e., data for naturally colored Early Roman Empire glass assemblages, were also reported in [3,5].

Two samples dated to the 4th c. AD, namely Histria-20 and Histria-39, were initially identified as HIMT (high iron manganese titanium) glass, according to their high iron and manganese content. In particular, sample Histria-20 can be assigned to the HIMTb sub-group; for details on this refined classification, see [61,62,63].

The term HIMT was coined by Freestone [59], though this type of glass was evidenced for the first time by Mirti and colleagues [64]. It is the equivalent of Foy *Groupe 1* [24]. This is an example of a Roman glass chemical type that appeared sometimes during the 4th c. AD, made using particular Egyptian sand, rich in iron-bearing minerals [63].

However, as stated above, according to its high magnesia (1.67 wt% MgO) and potash (1.77 wt% K_2_O), sample Histria-39 suggests a plant ash glass component in its recipe. Plant ash glass items were not frequently encountered in the zones under the Roman influence, where natron glass was the prevailing type of glass. An interesting overview about the occurrence of plant ash glass finds in the Roman world is given in [65]. The water content of this sample is also high (3.494 wt% H_2_O), possibly a consequence of the thick deposits of hydrated minerals still present on the surface of this fragment.

Several samples from the ACS Sector in Histria dated from the 4th c. onwards, namely Histria-34, Histria-35, Histria-36, and Histria-38, were identified as Foy *Série 2.1* [24]. The last two samples have relatively high amounts of H_2_O, which can be correlated with the thick layers of soil/weathering compound deposits covering their surfaces.

Sample Histria-37 is a special one. According to its high manganese and iron content, this pale blue handle of lamp dated to the 6th c. AD was included in the Foy *Série 2.1* group. However, its composition resembles that of RNCBGY2 glass, too, particularly considering its low Al_2_O_3_ and TiO_2_ concentrations, at least compared to the other Foy *Série 2.1* samples reported in this paper. Taking into account the 110 ppm CoO from its composition, we can infer that this fragment was produced by recycling, the manufacturing process involving cullet belonging to Foy *Série 2.1* and RNCBGY2 glass groups. Decorative elements made of intentionally colored blue glass might have accidentally entered the batch, thus the explanation for the presence of cobalt in minute amounts.

The presence of cobalt traces (100 ppm CoO) in the colorless glass fragment Tomis-2, a Mn-colorless sample, is also suggestive of recycling procedures. Co-containing glass might have been carelessly introduced into the batch (for example, fragments of vessels with blue decoration might have been included in the cullet), which would have contaminated the entire melt.

From the entire sample set, only fragments Histria-37 and Tomis-2 show unambiguous evidence of recycling, provided by the presence of small amounts of a chromophore trace-element (namely cobalt) in naturally colored and colorless glass, respectively. It is worth mentioning here the detection limits for CoO of the PGAA setup, estimated to be 100 ppm.

Glass waste Tomis-1, a fragment found in a glass kiln, is another example of recycled sample, as this green piece of glass has a compositional pattern resembling that of Foy *Série 2.1*, RNCBGY2, and colorless glass samples.

Compositionally speaking, glass working waste fragments Tomis-45, Tomis-46, and Tomis-48, fragments of molten glass with tiny pieces of wattle from the kiln walls attached on their surfaces, are clear outliers. A possible interpretation of these usual compositional patterns is that these chunks resulted from recycling many types of glass. In particular, Tomis-45 and Tomis-48 are somehow compositionally similar, featuring remarkably high alumina content (6.23 and 7.46 wt% Al_2_O_3_, respectively). Considering the composite structure of these samples (photos of these particular items were published in [44], one might interpret these results as stemming from the alumino-silicates compounds (wattle accretions) that are still present on their surfaces, resulting in the relatively high concentrations of alumina. Further analyses using optical microscopy and scanning electron microscopy (SEM) might be performed in the future on these particular samples to clarify these results, taking into account the fact that the PGAA technique provides the bulk chemical composition of the analyzed object.

Histria-31, a fragment from a colorless beaker, is also an interesting sample. Its color and its overall compositional pattern did not allow its assignation to any of the colorless/intentionally decolorized glass groups, as it contains antimony and manganese in small amounts. Moreover, its chemical composition does not present any similarity with RNCBGY2 glass. Dated on archaeological grounds to 4th–5th c. AD, it was identified as a Foy *Série 3.2* glass [24].

Tomis-2 and Tomis-3 are both fragments from vessels with spherical bodies, long necks, and flaring mouths, similar not only in shape but also in dimensions; both items were discovered in graves. Tomis-2 is colorless, while Tomis-3 has a yellow tinge. According to archaeological arguments, these containers were considered as local products; see the Introduction. Chemically speaking, Tomis-2 turned out to be Mn-colorless glass, while Tomis-3 was assigned to the RNCBGY2 group, i.e., it has a composition typical for the Early Roman glass.

Tomis-4 and Tomis-5 belong to the same type of colorless flask; they originated from the same context, i.e., the same grave, being dated to the 2nd–3rd c. AD. Both items were made of Sb–Mn colorless glass involving the recycling and mixing of two different chemical types of intentionally decolorized glass.

Tomis-8 and Tomis-9 are fragments from the same type of bulbous *unguentarium* (Isings 105 form) dated to the 4th c. AD. Both fragments are colorless, but Tomis-8 has olive tinges and Tomis-9 has green tinges. Both of them were identified as Mn-colorless glass.

Histria-16 and Histria-32 are fragments of square bottles, a well-known type of container from the 1st–2nd c. AD (Isings 50 form). Histria-16 has a pale-blue color, and Histria-32 has green tinges. Histria-16 was found in the BFL Sector and Histria-32 in the ACS Sector. Histria-16 was identified as RNCBGY2 glass/typical Early Roman glass, while Histria-32 is Sb–Mn colorless glass.

Two fragments of plates or shallow bowls were found in the BFL Sector, Histria-21 and Histria-27, both with blue-green tinges. They were made of RNCBGY2 glass, i.e., typical Early Roman glass of Levantine origin.

Histria-35 and Histria-36 are fragments of Late Roman Empire goblets (Isings 111) that were discovered in the ACS Sector. Both samples belong to Foy *Série 2.1*. A relatively large number of similar finds were also identified at Tropaeum Traiani, a site relatively close to both Histria and Tomis [16].

Histria-43 and Histria-44 are fragments of some Late Roman Empire goblets, found in the ACS Sector; both are Mn-colorless glass.

We tried to compare the composition of the glass samples unearthed in the BFL and ACS Sectors, the two particular places where the Histria fragments analyzed in this study were found. Thus, most of the samples from the BFL Sector were assigned to the RNCBGY2 group. The following exceptions occurred: Histria-23 bead, dated to the 2nd–3rd c. AD, assigned to Mn-colorless glass group; the fragment of a honeycomb cup Histria-20, dated to the 4th c. AD, attributed to the HIMT group; and the flask fragment Histria-30, pertaining to the Sb–Mn colorless glass group. However, all samples originating from the ACS Sector belong to several compositional groups discussed in the text: Foy *Série 2.1* and *Série 3.2*, Sb-colorless, Mn-colorless, and Sb–Mn colorless. Remarkably, a single RNCBGY2 sample was identified among those discovered at the ACS Sector, namely Histria-40. This might be explained by the fact that many of the ACS fragments chosen for this study turned out to be dated slightly later than those from the BFL Sector. Thus, the BFL Sector provided most of the Early Roman Empire samples *stricto sensu* (i.e., dated until the 4th c. AD), and, in turn, most of the BFL samples were included in the RNCBGY2 group.

Regarding the Tomis samples, most of the Sb–Mn colorless samples are from this site and none of them was identified as HIMT, Foy *Série 2.1*, or Foy *Série 3.2*, i.e., none of them pertains to any Late Roman Empire glass groups. In accordance with their archaeological and stylistic dating, the glass fragments discovered at Tomis proved to have compositional patterns typical for the Early Roman Empire period.

The Roman glass samples (15 fragments from the RNCBGY2 group) were shaped from glass fused in primary workshops located on the Levantine coast of the Mediterranean Sea [26]. The relatively small concentrations of titanium and iron oxides (0.07 wt% TiO_2_ and 0.35 wt% Fe_2_O_3_ on average) suggest the use of mature sand in their making.

According to the literature data [13,63], the primary glass of the Sb-colorless glass, of some of the Mn-colorless fragments, as well as of the Late Roman Empire items, those belonging to HIMT, Foy *Série 2.1*, and Foy *Série 3.2* groups, was most likely produced in Egyptian primary workshops.

Of clear interest is the composition of the glass working waste fragments discovered in the excavations from Tomis reported in this paper. It turned out that they are clear outliers compared to the rest of the glass samples published here, which are mostly vessel fragments. New questions were triggered by the bulk PGAA results that might be only answered by further analyses using some microscopy techniques involving minimally invasive sampling of these artefacts. In any case, the composition of these items suggests recycling procedures, having as input various types of glass, certainly a feature of the glass manufacturing in the secondary workshops from Tomis during the Early Roman Empire period.

The recycling of many types of decolorized glass is also evidenced in the nine Sb–Mn colorless glass samples from this assemblage.

Other samples resulting from glass recycling are two naturally colored fragments, Tomis-2 and Histria-37 that contain cobalt in minute amounts, as well one in Histria-39 sample, whose composition indicates the use of plant ash cullet in its making.

Based on the PGAA data reported in this paper, we can speculate that glass recycling procedures were used in the manufacturing of the vitreous finds discovered at Histria and Tomis.

This study indicates that both vessels made of fresh glass imported from the Mediterranean region as well as containers made of recycled glass, the latter ones probably locally manufactured in workshops active at Tomis, were used by the inhabitants of Histria and Tomis during the Roman period.

It is worth stressing a methodological conclusion, namely that all glass samples reported in this paper, exclusively analyzed using PGAA technique, were relatively easy assigned to well-known chemical groups from the archaeometric literature, allowing straightforward comparisons with coeval finds from other sites from all over the Roman Empire and further speculations on provenance and manufacturing practices.

## 5. Conclusions

This publication reports the composition of 48 glass fragments discovered during archaeological excavations at Histria and Tomis, Romania, mainly dated to the Early Roman Empire period. PGAA data allowed the attribution of the analyzed glass fragments to several well-established glass types from the archaeometric literature: Sb-colorless, Mn-colorless, Sb–Mn colorless, and RNCBGY2. Several finds from the Late Roman Empire period were identified as being made of HIMT, Foy *Série 2.1*, and Foy *Série 3.2* glass.

Using the PGAA data, clues for glass recycling were evidenced in several samples. Archaeological and compositional arguments support the idea that during the Early Roman Empire period, recycling practices were in use in the secondary glass workshops from Tomis.

The reported glass finds, discovered in archaeological excavation at Histria and Tomis, mostly dated according to typological and stratigraphic criteria to the Early Roman Empire period, were manufactured from raw glass fused in primary workshops in both Egypt and on the Levantine coast of the Mediterranean Sea. Moreover, PGAA data bring additional circumstantial evidence for the hypothesis of secondary local glass working in Tomis, glass manufacturing of different types of vessels involving the use of various types of cullet.

This study contributes to the understanding on life during the Roman time in two cities on the western shore of the Black Sea: Histria and Tomis. An impressive number of different glass vessels with various chemical compositions were used in everyday life, as well as in the burial rituals, offering a better perception on the glass consumption mechanisms during the Roman period in this zone. This publication brings details on the circulation of this fascinating material in the Black Sea area, harmoniously integrating this region within the landscape of archaeometric publications on Roman glass.

## Data Availability

All the data were published in this paper; the tables are in the supplementary material available at the link indicated above.

## Supplementary Materials

The following supporting information can be downloaded at: https://www.mdpi.com/article/10.3390/ma15020403/s1. Table S1 Archaeological description of the investigated glass samples [29,30,39,44,66,67,68,69,70,71,72,73,74,75,76,77,78,79,80,81,82]. Table S2 PGAA data for Brill B and Brill C archaeological reference glasses from Corning Museum of Glass [52] and published values [54]. Table S3 PGAA data for Histria and Tomis glass samples expressed in oxide wt% (except for Cl that is given in elemental form). Blank cells mean below the detection limits of the PGAA setup that are given in the second line of the same table. Samples were grouped according to their chemical composition; the mean values of the larger groups are provided, too. The corresponding comparison terms from the literature are also shown.

## References

[1] Shortland A.J., Rehren T., Richards M.P., Britton K. (2020). Glass. Archaeological Science—An Introduction.

[2] Freestone I.C., Klimscha F. (2021). Glass production in the first millennium CE: A compositional perspective. Ancient Glass and Glass Production, Edition TOPOI.

[3] Nenna M.D., Vichy M., Picon M. (1997). L’atelier de verrier de Lyon du 1er siècle après J.-C. et l’origine des verres “romains”. Revue d’Archéométrie.

[4] Sayre E.V., Smith R.W. (1961). Compositional categories of ancient glass. Science.

[5] Silvestri A., Gallo F., Maltoni F., Degryse P., Ganio M., Longinelli A., Molin G., Rosenow D., Phelps M., Meek A., Freestone I.C. (2018). Things that Travelled: A Review of the Roman Glass from Northern Adriatic Italy in Things That Travelled. Mediterranean Glass in the First Millennium AD.

[6] Gorin-Rosen Y., Nenna M.D. (2000). The ancient glass industry in Israel: Summary of the finds and new discoveries. La route du Verre: Ateliers Primaires et Secondaires du Second Millénaire Av J-C au Moyen Âge.

[7] Nenna M.D., Bayley J., Freestone I.C., Jackson C.M. (2015). Primary glass workshops in Graeco-Roman Egypt: Preliminary report on the excavations of the site of Beni Salama, Wadi Natrun (2003, 2005–2009). Glass of the Roman World.

[8] Tal O., Jackson-Tal R.E., Freestone I.C. (2004). New evidence of the production of raw glass at late Byzantine Apollonia-Arsuf, Israel. J. Glass Stud..

[9] Fontaine S.D., Foy D. (2007). L’épave Ouest-Embiez 1, Var: Le commerce maritime du verre brut et manufacturé en Méditerranée occidentale dans l’Antiquité. Rev. Archéol. Narbonn..

[10] Thirion-Merle V., Nenna M.D., Picon M., Vichy M. (2002–2003). Un Nouvel Atelier Primaire Dans le Wadi Natrun (Egypte) et les Compositions des Verres Produits Dans Cette Région.

[11] Silvestri A., Molin G., Salviulo G. (2008). The colourless glass of *Iulia Felix*. J. Archaeol. Sci..

[12] Freestone I.C. (2015). The recycling and reuse of Roman glass: Analytical approaches. J. Glass Stud..

[13] Gliozzo E. (2017). The composition of colourless glass: A review. Archaeol. Anthropol. Sci..

[14] Bugoi R., Alexandrescu C.G., Panaite A. (2018). Chemical composition characterization of ancient glass finds from *Troesmis—*Turcoaia, Romania. Archaeol. Anthropol. Sci..

[15] Bugoi R., Talmaţchi G., Szilágyi V., Harsányi I., Cristea-Stan D., Boţan S., Kasztovszky Z. (2021). PGAA analyses on Roman glass finds from Tomis. Rom. J. Phys..

[16] Bugoi R., Panaite A., Alexandrescu C.G. (2021). Chemical analyses on Roman and Late Roman Empire glass finds from the Lower Danube: The case of Tropaeum Traiani. Archaeol. Anthropol. Sci..

[17] Bugoi R., Ţârlea A., Szilágyi V., Harsányi I., Cliante L., Kasztovszky Z. (2022). Colour and beauty at the Black Sea coast: Archaeometric analyses of selected small finds from Histria. Rom. Rep. Phys.

[18] Stawiarska T. (2014). Roman and Early Byzantine Glass from Romania and Northern Bulgaria. Archaeological and Technological Study.

[19] Bugoi R., Poll I., Mănucu-Adameşteanu G., Neelmeijer C., Eder F. (2013). Investigations of Byzantine glass bracelets from Nufăru, Romania using external PIXE-PIGE methods. J. Arch. Sci..

[20] Bugoi R., Poll I., Manucu-Adamesteanu G., Calligaro T., Pichon L., Pacheco C. (2016). PIXE-PIGE analyses of Byzantine glass bracelets (10th–13th centuries AD) from Isaccea, Romania. J. Radioanal. Nucl. Chem..

[21] Bugoi R., Poll I., Mănucu-Adameşteanu G., Pacheco C., Lehuédé P. (2018). Compositional study of Byzantine glass bracelets discovered at the Lower Danube. Microchem. J..

[22] Bugoi R., Măgureanu A., Măgureanu D., Lemasson Q. (2020). IBA analyses on glass beads from the Migration Period. Nucl. Instrum. Method B.

[23] Bugoi R., Mureşan O. (2021). A brief study on the chemistry of some Roman glass finds from Apulum. Rom. Rep. Phys..

[24] Foy D., Picon M., Vichy M., Thirion-Merle V., Foy D., Nenna M.D. (2003). Caractérisation des verres de la fin de l’Antiquité en Mediterranée occidentale: l’émergence de nouveaux courants commerciaux. Échanges et Commerce du Verre dans le Monde Antique. Actes du Colloque de l’AIHV, Aix-en-Provence et Marseille, Juin 2001. Monographies Instrumentum 24.

[25] Foy D., Thirion-Merle V., Vichy M. (2004). Contribution à l’étude des verres antiques décolorés à l’antimoine. Revue d’Archéométrie.

[26] Gliozzo E., Turchiano M., Giannetti F., Santagostino Barbone A. (2016). Late Roman Empire glass vessels and production indicators from the town of *Herdonia* (Foggia, Italy): New data on CaO-rich/weak HIMT glass. Archaeometry.

[27] Condurachi E. (1954). Histria. Monografie Arheologică Vol. I.

[28] Condurachi E. (1966). Histria. Monografie Arheologică Vol. II.

[29] Băjenaru C., Bâltâc A. (2000–2001). Depozitul de candele de sticlă descoperit la basilica episcopală de la Histria. Pontica.

[30] Băjenaru C., Bâltâc A. (2006). Histria—Basilica Episcopală. Catalogul descoperirilor de sticlă (1984–2000). Pontica.

[31] Boţan S.P. (2015). Vase de Sticlă în Spaţiul dintre Carpaţi şi Prut (Secolele II a.Chr.–II p.Chr.).

[32] Chiriac C., Boţan S.P., Panait Bîrzescu F., Birzescu I., Matei-Popescu F., Robu K. (2013). Sticlăria elenistică şi romană din Pontul Euxin. Între producţie şi import. Poleis în Marea Neagră. Relaţii Interpontice şi Producţii Locale, Pontica et Mediterranea I.

[33] Țârlea A., Cliante L. (2020). ‘Put the lights on’: Early Byzantine stemmed goblets and lamps from the Acropolis Centre-South Sector in Histria (I). Peuce SN.

[34] Achim I., Bottez V., Angelescu M., Cliante L., Țârlea A., Lițu A. (2021). A city reconfigured: Old and new research concerning Late Roman urbanism in Istros. The Greeks and Romans in the Black Sea and the Importance of the Pontic Region for the Graeco–Roman World (7th Century BC–5th Century AD): 20 Years On (1997–2017); Tsetskhladze, G.R., Avram, A., Hargrave, J., Eds.; In Proceedings of the Sixth International Congress on Black Sea Antiquities, Constanƫa, Romania, 18–22 September 2017, Dedicated to Prof. Sir John Boardman to Celebrate his Exceptional Achievement and His 90th Birthday.

[35] Bottez V., Liţu A., Ţârlea A. (2015). Preliminary results of the excavations at Histria. The Acropolis Centre-South Sector (2013–2014). MCA SN.

[36] Achim I. (2014). La basilique à crypte d’Istros: Dix campagnes de fouilles (2002–2013). MCA.

[37] Achim I., Dima M., Beldiman C., Surdu V., Băcăran M., Munteanu F. (2014). Comuna Istria, jud. Constanța. “Basilica cu criptă”. Cronica Cercetărilor Arheologice din România, Campania 2013.

[38] Bărbulescu M., Buzoianu L. (2012). Tomis. Comentariu Istoric și Arheologic.

[39] Bucovală M. (1968). Vase Antice de Sticlă la Tomis.

[40] Bucovală M. (1969). Tradiţii elenistice în materialele funerare de epocă romană timpurie la Tomis. Pontice.

[41] Bucovală M. (1984). Roman glass vessels discovered in Dobrudja. J. Glass Stud..

[42] Drăghici C., Drăghici C., Drăghici C. (2012). Glassware from Tomis. Chronological and typological aspects. Annales AIHV Thessaloniki 2009.

[43] Lungu V., Chera C. (1992). Importuri de vase de sticlă suflate în tipar descoperite în necropolele Tomisului. Pontica.

[44] Cliante L., Ţârlea A. (2020). Secondary glass kilns and local glass production in Tomis during the Roman times. CICSA J..

[45] Szentmiklósi L., Belgya T., Révay Z., Kis Z. (2010). Upgrade of the prompt gamma activation analysis and the neutron-induced prompt gamma spectroscopy facilities at the Budapest research reactor. J. Radioanal. Nucl. Chem..

[46] Révay Z., Molnár G.L. (2003). Standardisation of the prompt gamma activation analysis method. Radiochim. Acta.

[47] Yonezawa C., Molnár G.L. (2004). Quantitative analysis. Handbook of Prompt Gamma Activation Analysis with Neutron Beams.

[48] Révay Z., Belgya T., Kasztovszky Z., Weil J.L., Molnar G.L. (2004). Cold neutron PGAA facility at Budapest. Nucl. Instrum. Method B.

[49] Révay Z. (2009). Determining elemental composition using Prompt γ Activation Analysis. Anal. Chem..

[50] Constantinescu B., Cristea-Stan D., Szőkefalvi-Nagy Z., Kovács I., Harsányi I., Kasztovszky Z. (2018). PIXE and PGAA—Complementary methods for studies on ancient glass artefacts from Byzantine, Late Medieval to modern Murano glass. Nucl. Instrum. Method B.

[51] Kasztovszky Z., Kunicki-Goldfinger J.J., Dzierżanowski P., Nawrolska G., Wawrzyniak P. (2005) PGAA and EPMA as complimentary nondestructive methods for analysis of boron content in historical glass. Proceedings of the Art’05–8th International Conference on Non Destructive Investigations and Microanalysis for the Diagnostics and Conservation of the Cultural and Environmental Heritage.

[52] Moropoulou A., Zacharias N., Delegou E.T., Maróti B., Kasztovszky Z. (2016). Analytical and technological examination of glass tesserae from Hagia Sophia. Microchem. J..

[53] Zacharias N., Kaparou M., Oikonomou A., Kasztovszky Z. (2018). Mycenaean glass from the Argolid, Peloponnese, Greece: A technological and provenance study. Microchem. J..

[54] Adlington L.W. (2017). The Corning archaeological reference glasses: New values for “old” compositions. Pap. Inst. Archaeol..

[55] Révay Z. (2006). Calculation of uncertainties in Prompt Gamma Activation Analysis. Nucl. Instrum. Method A.

[56] Schreiner M., Woisetchlaeger G., Schmitz I., Wadsak M. (1999). Characterisation of surface layers formed under natural environmental conditions on medieval stained glass and ancient copper alloys using SEM, SIMS and atomic force microscopy. J. Anal. At. Spectrom..

[57] Shortland A.J., Schachner L., Freestone I., Tite M. (2006). Natron as a flux in the early vitreous materials industry: Sources, beginnings and reasons for decline. J. Archaeol. Sci..

[58] Al-Bashaireh K., Al-Mustafa S., Freestone I.C., Al-Housan A.Q. (2016). Composition of Byzantine glasses from Umm el-Jimal, northeast Jordan: Insights into glass origins and recycling. J. Cultural Herit..

[59] Freestone I.C., Hurst H.R. (1994). Appendix: Chemical analysis of “raw” glass fragments. Excavations at Carthage II, 1. The Circular Harbor, the Site and Finds Other Than Pottery.

[60] Brill R.H., Weinberg G.D. (1988). Scientific investigations of the Jalame glass and related finds. Excavations at Jalame: Site of a Glass Factory in Late Roman Palestine.

[61] Ceglia A., Cosyns P., Nys K., Terryn H., Thienpont H., Meulebroeck W. (2015). Late Roman Empire glass distribution and consumption in Cyprus: A chemical study. J. Archaeol. Sci..

[62] De Juan Ares J., Schibille N., Molina Vidal J., Sanchez de Prado M.D. (2019). The supply of glass at *Portus Ilicitanus* (Alicante, Spain): A meta-analysis of HIMT glasses. Archaeometry.

[63] Freestone I.C., Degryse P., Lankton J., Gratuze B., Schneider J., Rosenow D., Phelps M., Meek A., Freestone I. (2018). HIMT, glass composition and commodity branding in the primary glass industry. Things That Travelled, Mediterranean Glass in the First Millenium CE.

[64] Mirti P., Casoli A., Appolonia L. (1993). Scientific analysis of Roman glass from Augusta Praetoria. Archaeometry.

[65] Drauschke J., Greiff S. (2010). Chemical aspects of Byzantine glass from Caričin Grad/Iustiniana Prima (Serbia). Glass in Byzantium: Production, Usage, Analyses: International Workshop Organised by the Byzantine Archaeology Mainz, 17–18 of January 2008.

[66] Adam-Veleni P. (2010). Glass Cosmos.

[67] Antonaras A. (2012). Fire and Sand. Ancient Glass in the Princeton University Art Museum.

[68] Arveiller-Dulong V., Arveiller J. (1985). La Verre D’epoque Romaine au Musée Archéologique de Strasbourg.

[69] Arveiller-Dulong V., Nenna M.D. (2005). Les Verres Antiques du Musée du Louvre, Vol. II: Vaisselle et Contenants du Ier Siècle au Début du VIIe Siècle Après J.-C., Musée du Louvre.

[70] Atila C., Gürler B., Özerler M., Ünsalan D. (2009). Glass Objects from Bergama Museum/Bergama Muzesi Cam Eserleri.

[71] Buljević Z., Foy D., Nenna M.D. (2011). Imprints on the bottoms of glass bottles from Dalmatia held in the Archaeological Museum in Split. Corpus des Signatures et Marques sur Verres Antiques.

[72] Foy D. (2010). Les Verres Antiques d’Arles.

[73] Gorin-Rosen Y. (2015). The glass finds from Horbat Zefat ‘Adi (east). Hadashot Arkheologiyot.

[74] Gorin-Rosen Y. (2020). Glass finds and remains of a glass industry from Miska. Atiqot.

[75] Gorin-Rosen Y., Jackson-Tal R., Tzaferis V., Israeli S. (2008). Chapter 9: Area F: The glass finds. Paneas I, IAA Reports 37.

[76] Hayes J.W. (1975). Roman and Pre-Roman Glass in the Royal Ontario Museum: A Catalogue.

[77] Israeli Y. (2003). Ancient Glass in the Israel Museum.

[78] Lightfoot C.S. (2007). Ancient Glass in National Museums Scotland.

[79] Lightfoot C.S. (2017). Cesnola Collection of Cypriot Art.

[80] Lightfoot C.S., Arslan M. (1992). Ancient Glass of Asia Minor.

[81] Whitehouse D. (1997). Roman Glass in the Corning Museum of Glass.

[82] Whitehouse D. (2001). Roman Glass in the Corning Museum of Glass.

